# SC79 protects retinal pigment epithelium cells from UV radiation *via* activating Akt-Nrf2 signaling

**DOI:** 10.18632/oncotarget.11164

**Published:** 2016-08-09

**Authors:** Yi-qing Gong, Wei Huang, Ke-ran Li, Yuan-yuan Liu, Guo-fan Cao, Cong Cao, Qin Jiang

**Affiliations:** ^1^ The Affiliated Eye Hospital of Nanjing Medical University, Nanjing, China; ^2^ Ophthalmology Department, Zhenjiang First People's Hospital, Zhenjiang, China; ^3^ Institute of Neuroscience, Soochow University, Suzhou, Jiangsu, China

**Keywords:** retinal pigment epithelium, UV, SC79, Akt, Nrf2 signaling

## Abstract

Excessive Ultra-violet (UV) radiation causes oxidative damages and apoptosis in retinal pigment epithelium (RPE) cells. Here we tested the potential activity of SC79, a novel small molecule activator of Akt, against the process. We showed that SC79 activated Akt in primary and established (ARPE-19 line) RPE cells. It protected RPE cells from UV damages possibly via inhibiting cell apoptosis. Akt inhibition, via an Akt specific inhibitor (MK-2206) or Akt1 shRNA silence, almost abolished SC79-induced RPE cytoprotection. Further studies showed that SC79 activated Akt-dependent NF-E2-related factor 2 (Nrf2) signaling and inhibited UV-induced oxidative stress in RPE cells. Reversely, Nrf2 shRNA knockdown or S40T mutation attenuated SC79-induced anti-UV activity. For the *in vivo* studies, we showed that intravitreal injection of SC79 significantly protected mouse retina from light damages. Based on these results, we suggest that SC79 protects RPE cells from UV damages possibly via activating Akt-Nrf2 signaling axis.

## INTRODUCTION

Excessive Ultra-violet (UV) radiation and reactive oxygen species (ROS) are known as the important contributors of age-related macular degeneration (AMD) and other retinal degenerative diseases [1–3]. Retinal pigment epithelium (RPE) dysfunction, along with photoreceptor damages, and Bruch's membrane thickening, were observed in many AMD patients [1–3]. Our group and others have demonstrated that UV radiation will lead to ROS production and RPE cell oxidative stresses [4–6]. The latter will induce RPE cell apoptosis [4–8].

Akt is a well-established pro-survival signaling molecule [9]. Many stimuli were shown to activate Akt and inhibit RPE cell damages. For example, we showed that 3H-1,2-dithiole-3-thione (D3T) protected RPE cells from UV via activation of Akt signaling [6]. Further, Salvianolic acid A activated Akt cascade and inhibited ROS-induced RPE cell apoptosis [10].

A recent study by Jo et al., has characterized a novel, selective, and cell-permeable small molecule Akt activator: SC79 [11]. SC79 uniquely suppresses Akt membrane translocation while activating Akt in the cytosol [11]. SC79 has shown cytoprotective effects in experimental settings [11]. For example, SC79 could suppress excitotoxicity and alleviate stroke-induced neuronal death [11]. When given *in vivo,* it also protected against early brain injuries by subarachnoid hemorrhage [12]. Yet, Moreira et al., showed that activation of Akt by SC79 failed to reduce ischemic injury of the rat heart [13]. In the current study, we investigated its role on UV-induced RPE cell damages.

The transcriptional factor NF-E2-related factor 2 (Nrf2) dictates the transcription of key anti-oxidant genes [14, 15]. Activated Nrf2 enters the nucleus and binds to antioxidant-responsive element (ARE), causing transcription of several key anti-oxidant genes, including heme oxygenase-1 (HO-1) and NAD(P)H: quinone oxidoreductase (NQO-1) as well asγ-glutamyl cystine ligase catalytic subunit (GCLC) and γ-glutamyl cystine ligase modifying subunit (GCLM) [16]. In the present study, we showed that SC79 protected RPE cells from UV radiation via activating Akt and its downstream Nrf2 signaling.

## RESULTS

### SC79 activates Akt and protects RPE cells from UV damages

**Figure 1:**
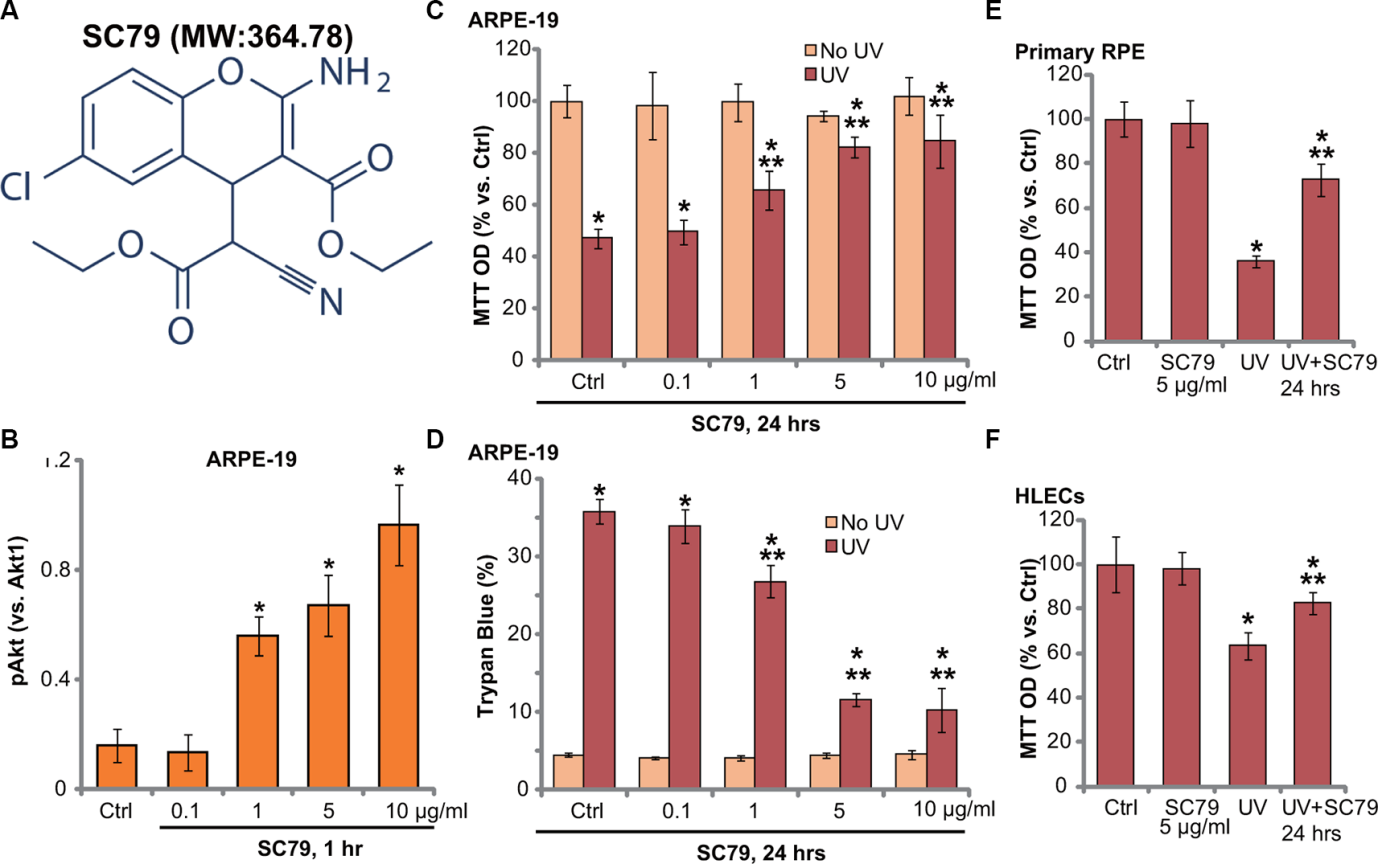
SC79 activates Akt and protects RPE cells from UV damages The molecular structure along with the molecular weight (MW) of SC79 were presented (**A**). ARPE-19 cells were treated with applied concentration of SC79 (0.1–10 μg/mL, or 2.74–27.41 μM) for 1 h, p-Akt (Ser-473) and Akt1 expression was tested by Western blots and was quantified (**B**) *n* = 4). ARPE-19 cells (**C** and **D**) primary murine RPE cells (“Primary RPE”, (**E)**) or HLECs (**F**) pretreated with applied concentration of SC79 for 30 min, were subjected to UV radiation (30 mJ/cm^2^), cells were further cultured for 24 h, and cell viability was tested by MTT assay (C, E and F); Cell death was detected by trypan blue staining assay (D). “Ctrl” stands for untreated control group (Same for all figures). For each assay, *n* = 5. Experiments in this figure were repeated three times to insure consistency of results. *p < 0.05 *vs.* “Ctrl” group (C–F). **p < 0.05 *vs.* UV only group (C–F).

The structure along with the molecular/formula weight of SC79 were presented in Figure 1A (see in [11, 13]). We tested whether the novel Akt specific activator could attenuate UV-induced RPE cell damages. We first demonstrated that SC79, at 1–10 μg/mL (2.74–27.41 μM), significantly activated Akt (p-Akt intensity increase) in ARPE-19 cells Figure 1B). Its activity on p-Akt was dose-dependent (Figure 1B). Notably, UV radiation-induced ARPE-19 cell viability reduction (MTT OD reduction, Figure 1C) and cell death (Trypan blue increase, Figure 1D) were largely inhibited by pretreatment of SC79 (1–10 μg/mL). The RPE cytoprotective activity by SC79 was also dose-dependent (Figure 1C and 1D). Since 5 μg/mL of SC79 displayed significant RPE-cytoprotective function (Figure 1C and 1D), this concentration was applied in following studies. Intriguingly, SC79 (at 5 μg/mL) also inhibited UV-induced viability reduction in primary murine RPE cells and in human lens cells (HLECs) (Figure 1E and 1F). Together, these results demonstrate that SC79 activates Akt and protects RPE cells from UV injuries.

### SC79 inhibits UV-induced apoptosis activation in RPE cells

**Figure 2:**
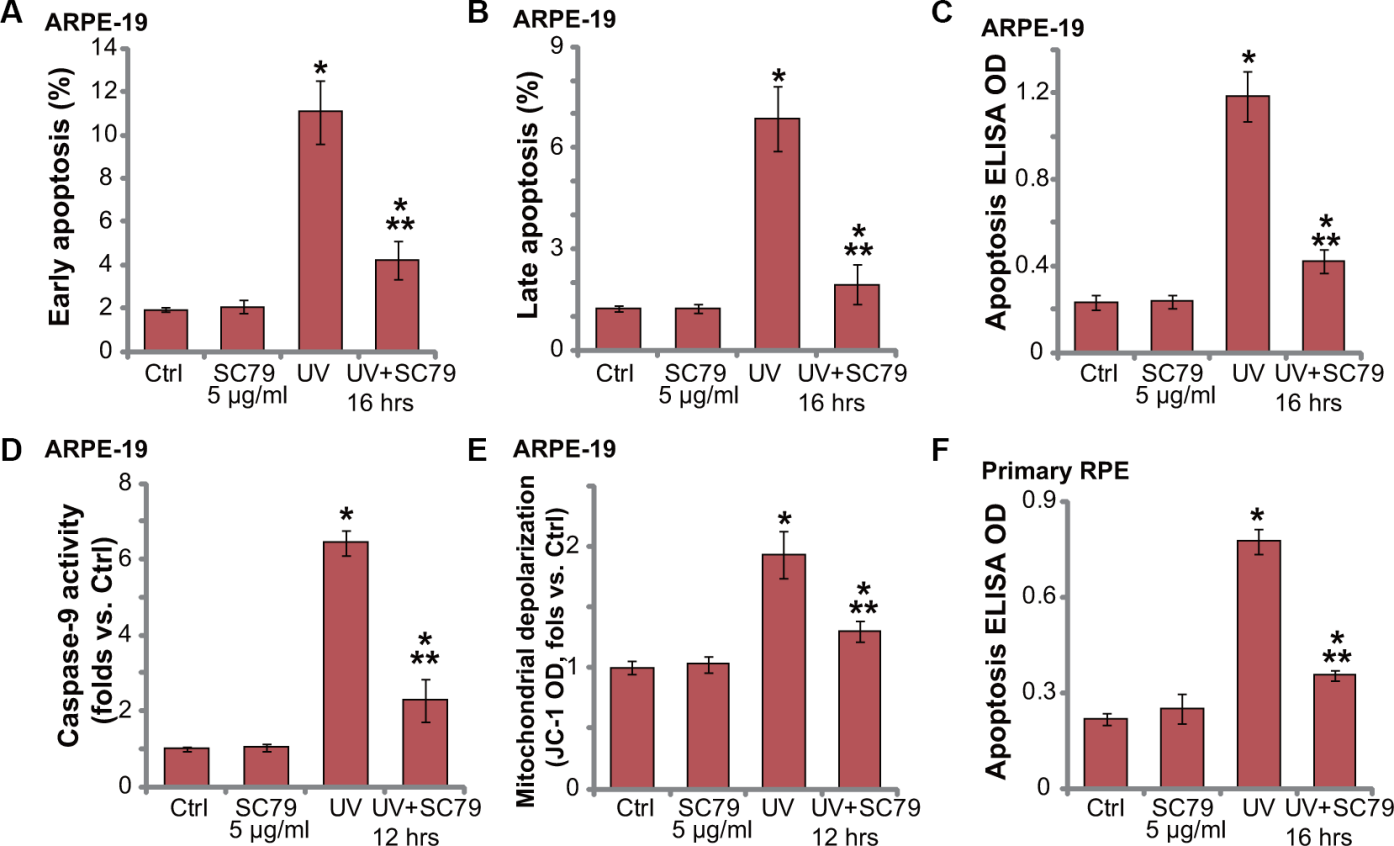
SC79 inhibits UV-induced apoptosis activation in RPE cells ARPE-19 cells (**A**–**E**) or primary murine RPE cells (“Primary RPE”,(**F**)) were pretreated with SC79 (5 μg/mL) for 30 min prior to UV radiation (30 mJ/cm^2^), cells were further cultured for applied time, and cell apoptosis was tested by listed assays (A–F). For each assay, *n* = 5. Experiments in this figure were repeated three times to insure consistency of results. *p < 0.05 *vs.* “Ctrl” group. **p < 0.05 *vs.* UV only group.

Our studies and others have shown that UV radiation induces RPE cell apoptosis [4, 5, 10, 17–20]. We thus wanted to know if SC79-mediated RPE cytoprotection was due to apoptosis inhibition. In line with our previous studies [4, 6], we showed that UV radiation induced significant apoptosis activation in ARPE-19 cells (Figure 2A–2C). Apoptosis activation by UV was tested by the Annexin V FACS assay (Figure 2A and 2B) and Histone DNA ELISA assay (Figure 2C). Significantly, pre-treatment with SC79 (5 μg/mL) dramatically attenuated UV-induced apoptosis activation in ARPE-19 cells (Figure 2A–2C).

Further studies showed that UV radiation also induced caspae-9 activation (Figure 2D) and mitochondrial depolarization (Figure 2E) in ARPE-19 cells, indicating mitochondrial-dependent apoptosis pathway activation by UV [21]. Such an effect in UV-radiated RPE cells was again largely inhibited by SC79 (5 μg/mL) pretreatment. Histone DNA ELISA results in primary murine RPE cells confirmed apoptosis activation in UV-irradiated primary cells (Figure 2F), which was again inhibited by SC79 pretreatment (Figure 2F). Together, these results demonstrate that SC79 inhibits UV-induced apoptosis activation in RPE cells.

### SC79-mediated RPE cytoprotection against UV requires Akt activation

**Figure 3:**
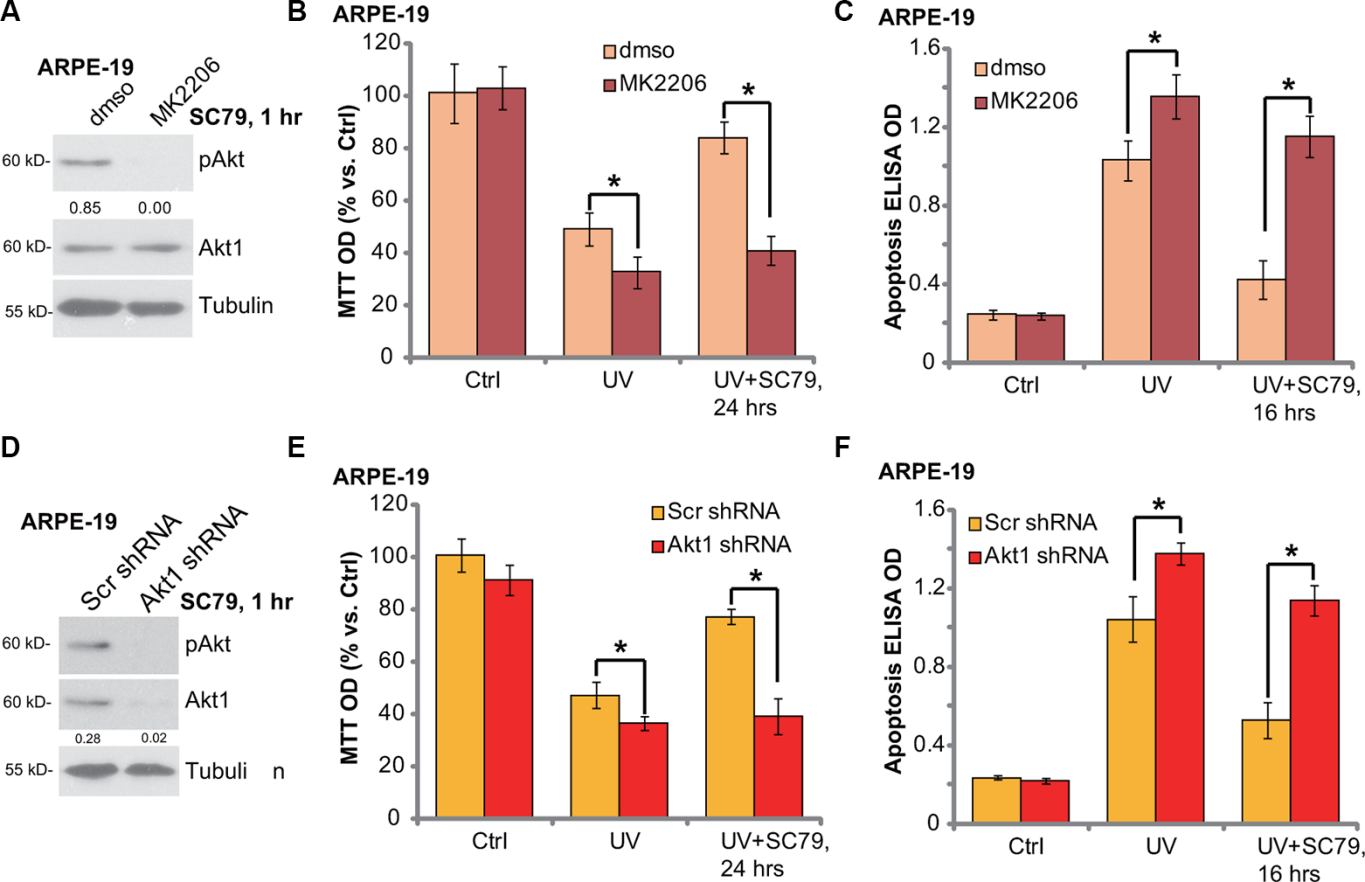
SC79-mediated RPE cytoprotection against UV requires Akt activation ARPE-19 cells were pre-treated with MK-2206 (5 μM) for 1h, followed by SC79 (5 μg/mL) treatment for 1 h, p-Akt (Ser-473) and Akt1 expression was tested by Western blot assay (**A**). ARPE-19 cells were pre-treated with MK-2206 (5 μM) for 1 h, followed by UV radiation (30 mJ/cm^2^), or plus SC79 (5 μg/mL, 30 min prior UV), cells were further cultured for applied time; Cell viability ((**B**) MTT assay) and cell apoptosis ((**C**) Histone DNA ELISA assay) were tested. The stably ARPE-19 cells expressing scramble control shRNA (“Scr shRNA”) or Akt1 shRNA were treated with UV (30 mJ/cm^2^) radiation, or plus SC79 (5 μg/mL, 30 min prior UV); Cells were further cultured for applied time, cell viability (**E**) and cell apoptosis (**F**) were tested. SC79 (5 μg/mL, 1 h)-induced Akt activation in above cells was tested by Western blot assay (D). Akt phosphorylation (vs. regular Akt1) was quantified (A), Akt1 expression (vs. Tubulin) was quantified (**D**). “dmso” stands for 0.1% DMSO vehicle control (B and C). For each assay, *n* = 5. Experiments in this figure were repeated three times to insure consistency of results. *p < 0.05.

To elucidate to link between Akt activation and SC79-induced RPE cytoprotection, we first applied an Akt specific inhibitor: MK-2206 [22]. SC79-induced Akt activation was completely blocked by MK-2206 (Figure 3A). MTT assay results in Figure 3B and apoptosis ELISA assay results in Figure 3C demonstrated that MK-2206 co-treatment potentiated UV damages in ARPE-19 cells, leading to profound cell death and apoptosis. More importantly, SC79-mediated RPE cytoprotection against UV was almost abolished with MK-2206 co-treatment (Figure 3B and 3C).

To rule out the possible off-target effect of MK-2206, we utilized targeted shRNA to selectively knockdown Akt1 in ARPE-19 cells (See the same protocol in our previous study [6]). SC79-induced Akt activation was almost blocked in Akt1-silenced cells (Figure 3D). These Akt1-shRNA expressing stable ARPE-19 cells were more vulnerable to UV radiation (Figure 3E and 3F). In line with pharmacological data, SC79 was unable to rescue ARPE-19 cells from UV when Akt1 was silenced (Figure 3E and 3F). Collectively, we suggest that SC79-induced RPE cytoprotection against UV requires Akt activation.

### SC79 activates Nrf2 signaling in RPE cells

**Figure 4:**
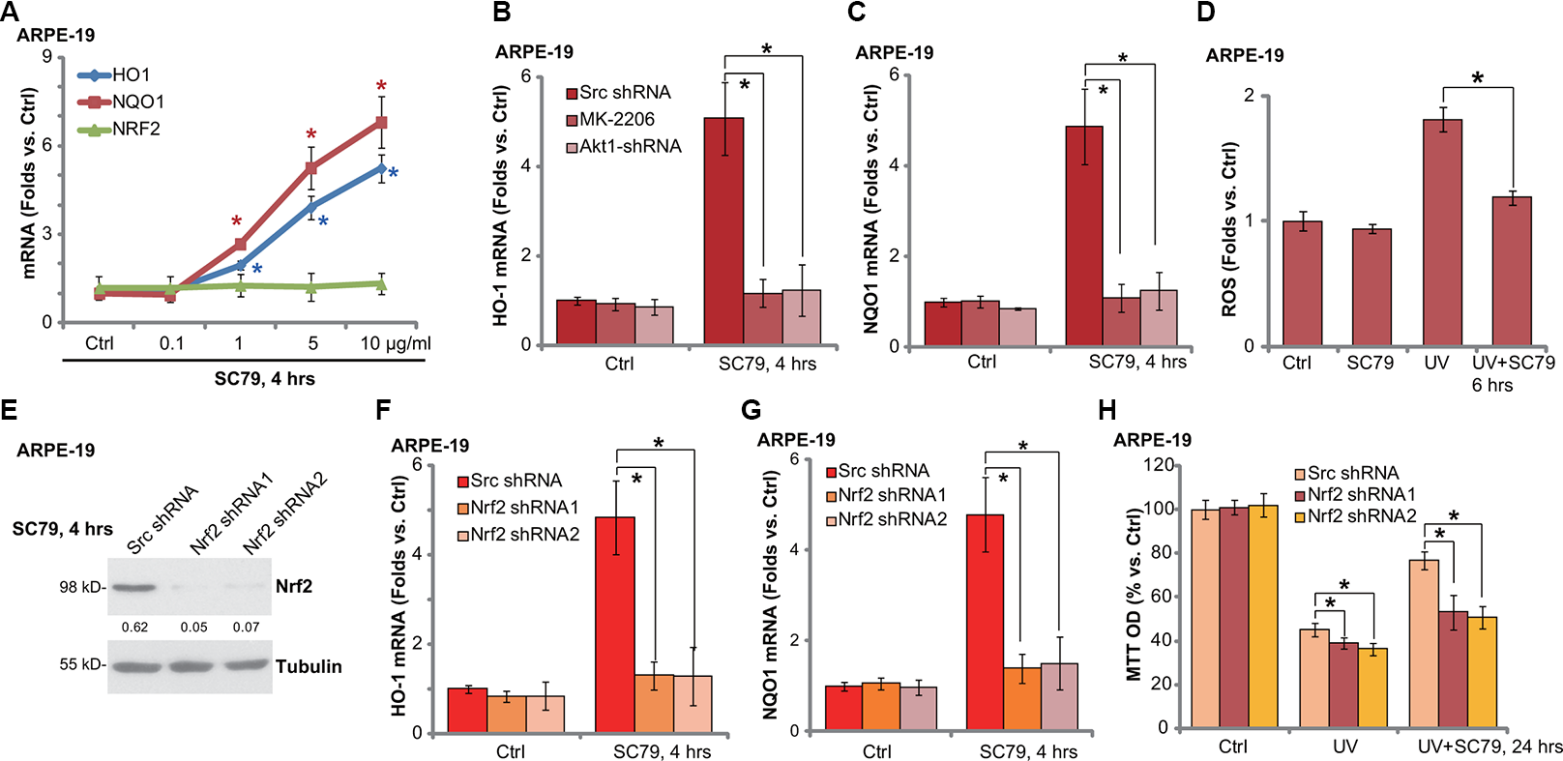
SC79 activates Nrf2 signaling in RPE cells ARPE-19 cells were treated with applied concentration of SC79 (0.1–10 μg/mL) for 4 h, mRNA expression of HO-1, NQO-1 and Nrf2 was tested by RT-qPCR assay (**A**). Stably ARPE-19 cells with scramble-shRNA (“Scr shRNA”) or Akt1 shRNA were treated with SC79 (5 μg/mL) or plus MK-2206 (5 μM), HO-1 and NQO-1 mRNA expression was tested by RT-qPCR assay (**B** and **C**). ARPE-19 cells, pretreated with SC79 (5 μg/mL) for 30 min, were subjected to UV radiation (30 mJ/cm^2^), cells were further cultured and relative ROS production was tested (**D**). The stably ARPE-19 cells with scramble-shRNA (“Scr shRNA”) or Nrf2 shRNA (“−1/−2”, with non-overlapping sequences) were treated with SC79 (5 μg/mL) for indicated time, expressions of listed proteins and mRNA were tested by Western blot assay (**E**) and RT-qPCR assay (**F** and **G**) respectively. Above cells were treated with UV (30 mJ/cm^2^) radiation, or plus SC79 (5 μg/mL, 30 min prior UV), cells were further cultured for 24h and cell viability was evaluated by MTT assay (**H**). HO-1 protein expression (vs. Tubulin) was quantified (E). For each assay, *n* = 5. Experiments in this figure were repeated three times to insure consistency of results. *p < 0.05 vs. “Ctrl” group (A). *p < 0.05 (B, C, E and F).

As described, activation of Nrf2 signaling may inhibit UV damages in RPE cells [6]. We therefore tested whether SC79 could also activate this signaling. Real-time quantitative PCR (RT-qPCR) assay results demonstrated that SC79 dose-dependently increased mRNA expression of Nrf2-regulated genes: HO-1 and NQO-1 (Figure 4A) [15]. Nrf2 mRNA expression itself was not changed following the SC79 treatment (Figure 4A). We also tested the expression of other Nrf2-regulated genes: γ-glutamyl cystine ligase catalytic subunit (GCLC) and γ-glutamyl cystine ligase modifying subunit (GCLM) [6]. Real-time PCR results in Supplementary Figure S1 showed that mRNA expression of GCLM and GCLC was significantly increased in SC79 (1–10 μg/mL)-treated ARPE-19 cells. Intriguingly, SC79-induced HO-1 (Figure 4B) and NQO-1 (Figure 4C) mRNA expression was almost blocked by MK-2206 or Akt1 shRNA, suggesting that activation of Akt is required for HO-1/NQO-1 expression by SC79. Notably, as shown in Figure 4D, UV-induced ROS production was also significantly alleviated with SC79 pretreatment.

To support the involvement of Nrf2 signaling in SC79-mediated RPE cytoprotection, we once again utilized targeted shRNA to knockdown Nrf2 in ARPE-19 cells. Two non-overlapping Nrf2 shRNAs were applied here [6], both of them significantly downregulated Nrf2 expression in ARPE-19 cells (Figure 4D). Notably, SC79-mediated HO-1 (Figure 4F) and NQO-1 (Figure 4G) mRNA expression was largely inhibited by the Nrf2 shRNAs. Significantly, Nrf2 shRNAs also largely attenuated SC79-mediated RPE cytoprotection (Figure 4H) and apoptosis inhibition (Data not shown). In line with our previous studies [6], Nrf2 shRNA knockdown again augmented UV damages in ARPE-19 cells (Figure 4H). These results imply that SC79 activates Akt-dependent Nrf2 signaling and protects RPE cells from UV-induced oxidative stresses.

### Nrf2 S40T mutation attenuates SC79-mediated RPE cytoprotection against UV radiation

**Figure 5:**
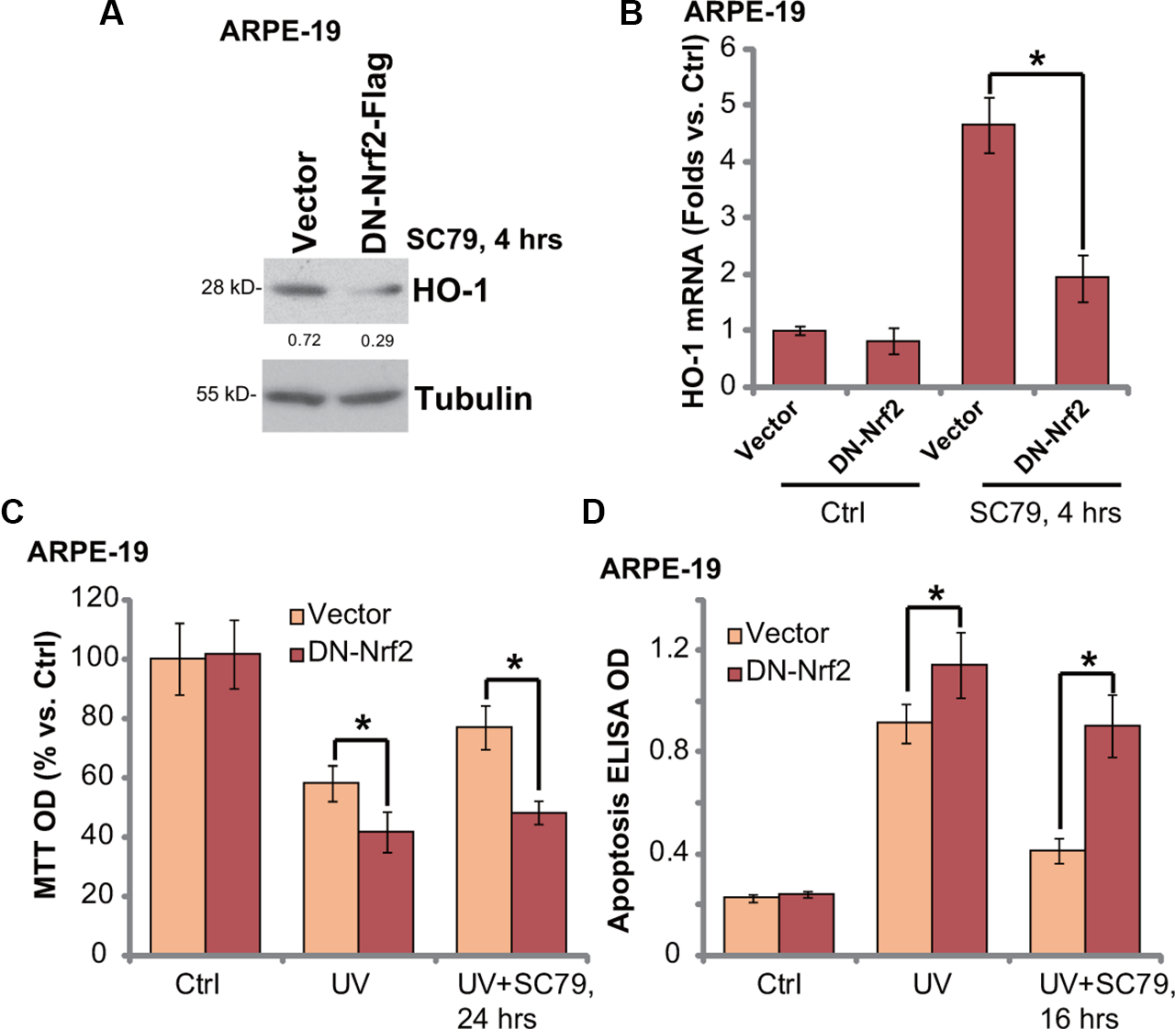
Nrf2 S40T mutation attenuates SC79-mediated RPE cytoprotection against UV radiation Stable ARPE-19 cells expressing dominant negative Nrf2 (S40T, “DN-Nrf2”, flag tagged) or empty vector (pSV2 puro Flag) were treated with SC79 (5 μg/mL) for indicated time, expressions of listed protein and mRNA were tested by Western blot assay (**A**) and RT-qPCR assay (**B**) respectively. Above cells were also subjected to UV (30 mJ/cm^2^) radiation, or plus SC79 (5 μg/mL, 30 min prior UV), cell viability and apoptosis were tested MTT assay (**C**) and Histone DNA ELISA assay (**D**) respectively. HO-1 protein expression (vs. Tubulin) was quantified (A). For each assay, *n* = 5. Experiments in this figure were repeated three times to insure consistency of results. *p < 0.05.

To further support the role of Nrf2 signaling in SC79-mediated RPE cytoprotection, a mutated dominant negative Nrf2 (DN, S40T) [6] was introduced to ARPE-19 cells. Significantly, SC79-induced HO-1 protein (Figure 5A) and mRNA (Figure 5B) expression was largely inhibited in DN-Nrf2-expressing cells. More importantly, SC79-mediated RPE cytoprotection, tested by viability recovery (Figure 5C) and apoptosis inhibition (Figure 5D), was largely attenuated in ARPE-19 cells with DN-Nrf2. DN-Nrf2 cells were again more sensitive to UV injuries (Figure 5C and 5D). Note that above Nrf2 shRNA or mutation didn't affect SC79-induced Akt activation in ARPE-19 cells (Data not shown). These results further suggest that activation of Nrf2 signaling is required for SC79-mediated RPE cytoprotection against UV.

### SC79 activates Nrf2 signaling in primary murine RPE cells

**Figure 6:**
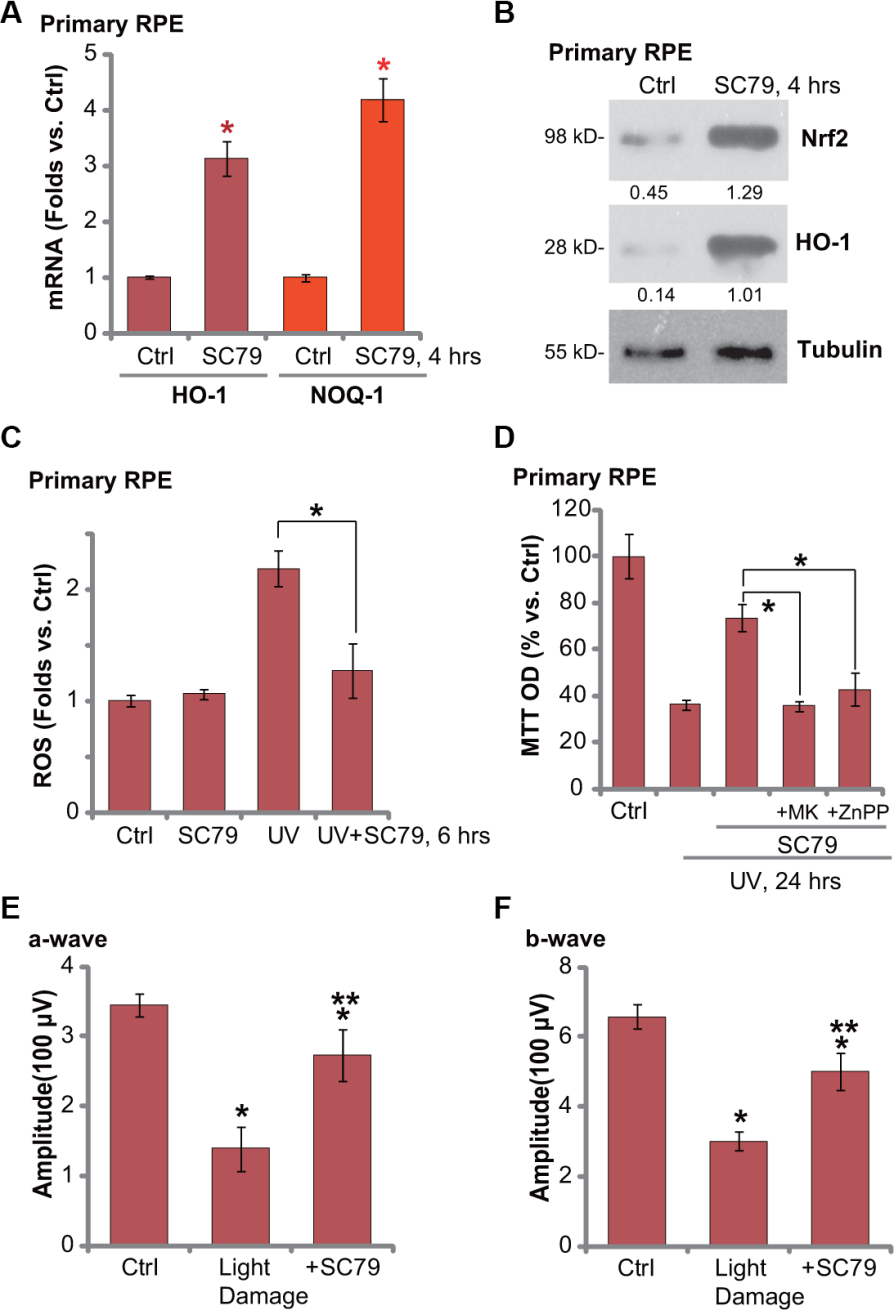
SC79 activates Nrf2 signaling in primary murine RPE cells and its retinal protection activity *in vivo* Primary murine RPE cells were treated with SC79 (5 μg/mL) for indicated time, expressions of listed mRNAs (**A**) and proteins (**B**) were tested. Primary murine RPE cells were treated with UV (30 mJ/cm^2^) radiation, or plus SC79 (5 μg/mL, 30 min prior UV), cells were further cultured before ROS content was analyzed (**C**). Primary murine RPE cells were pre-treated with SC79 (5 μg/mL), or plus MK-2206 (“MK”, 5 μM)/ZnPP (10 μM), followed by UV (30 mJ/cm^2^) radiation, cells were further cultured for 24 h before cell viability was tested (**D**). After the light exposure in mice retina, ERG was measured, quantified amplitudes of a- and b-waves were presented (**E** and **F**). For each assay, *n* = 5. *p < 0.05 vs. “Ctrl” group (A, E and F). **p < 0.05 vs. “Light damage” only group (E and F). *p < 0.05 (C and D).

We also tested Nrf2 signaling in SC79-treated primary murine RPE cells. As shown in Figure 6A, treatment of SC79 (at 5 μg/mL) induced mRNA expression of HO-1 and NQO-1 in primary murine RPE cells. Nrf2 mRNA expression was again not changed in SC79-treated primary cells (Data not shown). Further studies demonstrated Nrf2 protein accumulation and HO-1 protein expression in primary RPE cells with SC79 treatment (Figure 6B), further indicating Nrf2 activation. Intriguingly, UV-induced ROS production was again attenuated with SC79 pretreatment (Figure 6C). Significantly, SC79-mediated cytoprotection against UV in primary RPE cells was attenuated with co-treatment of the Akt inhibitor MK-2206 or the HO-1 inhibitor ZnPP [6] (Figure 6D). These results indicate that Akt-Nrf2 cascade activation is required for SC79-mediated cytoprotection against UV in primary RPE cells.

### SC79 intravitreal injection protects mouse retina from light damages

At last, the mouse retinal light damage model [6, 23] was applied to test the activity of SC79 *in vivo*. In line with our previous findings, twenty-four hours after the light exposure, retinal ERG a- and b-waves were markedly decreased (Figure 6E and 6F) [6]. Significantly, intravitreal injection of SC79 (50 ng/eye) largely attenuated above retinal changes (Figure 6E and 6F). SC79 administration alone failed to affected ERG in our system (Data not shown). These results indicate that SC79 intravitreal injection could protect mouse retina from light damages.

## DISCUSSION AND CONCLUSIONS

Excessive UV radiation will cause damages to RPE cells, which is an important contributor of AMD and other retinal diseases [1–3]. We here showed that SC79, a novel small molecular Akt activator [11, 12], activated Akt and protected established/primary RPE cells from UV injuries. SC79 largely attenuated UV-induced RPE cell apoptosis. Akt inhibition via the Akt specific inhibitor (MK-2206) or Akt1 shRNA almost abolished SC79-induced RPE cytoprotection against UV. Notably, we suggested that activation of Nrf2 signaling, as downstream of Akt, participated in SC79-mediated RPE cytoprotection against UV radiation.

Existing evidences have shown that Nrf2 activity could be modified by several upstream signaling axis, including Erk, p38 mitogen-activated protein kinases (MAPKs) and protein kinase C (PKC) [24]. More recent studies (including ours [6, 10]) have indicated that Akt and its downstream mTOR complex 1 (mTORC1) could also regulate Nrf2 activity. For example, a study by Lee et al., showed that sulforaphane activated Nrf2 through activating PI3K-Akt signaling [25]. Similarly, Xu et al., demonstrated that pyocyanin activated Nrf2 signaling downstream of PI3K-Akt [26]. Our previous studies showed that Salvianolic acid A-activated Nrf2-HO-1 signaling was dependent on Akt-mTORC1 signaling in RPE cells [10]. Recently, we showed that D3T protected RPE cells from UV radiation via activation of Akt downstream Nrf2-HO-1 signaling [6]. D3T induced Nrf2 phosphorylation at Ser-40 as downstream of Akt, which was required for subsequent Nrf2 activation [6]. In the current study, we showed that Nrf2 S40T mutation almost blocked SC79-mediated HO-1 expression as well.

Based on these results, we propose that SC79 activates Akt to possibly phosphorylate Nrf2 at Ser-40, causing Nrf2 accumulation and activation. As a matter of fact, SC79-induced Nrf2 signaling activation was almost blocked by the Akt inhibitor MK-2206 or Akt1 shRNA silence. Significantly, Nrf2 shRNA knockdown or S40T mutation dramatically attenuated SC79-induced RPE cytoprotection against UV. Therefore, SC79 activated Akt downstream Nrf2 to attenuate UV-induced oxidative stresses and RPE cell apoptosis. Importantly, the *in vivo* study results showed that intravitreal injection of SC79 offered a significant protection of mice retina against light damages. Thus, SC79 might have therapeutic values for treatment of AMD and other retinal degenerative diseases.

## MATERIALS AND METHODS

### Ethics

All methods listed in the study were carried out in accordance with the approved ethics guidelines of all authors' institutions.

### Reagents, chemicals and antibodies

MK-2206 (S1078) and SC79 (S7863) were from Selleck (Shanghai, China). The anti-β-tubulin antibody (SAB4500088) and the heme oxygenase-1 (HO-1) inhibitor Zinc protoporphyrin (ZnPP, 282820) were obtained from Sigma (St. Louis, MO). All other antibodies were from Cell Signaling Tech (Nanjing, China) and Santa Cruz Biotech (Shanghai, China) as described [6, 10].

### Cell culture

Culture of human retinal pigment epithelial cells (ARPE-19 line) and human lens epithelial cells (HLECs) were described previously [6, 27, 28]. The protocols of isolation and culture of primary murine RPE cells were described in our previous studies [6, 10]. The experiments were performed in accordance with the Institutional Animal Care and Use Committee (IACUC). The protocols were approved by all authors' institutions.

### UV radiation

UV radiation (UVB and UVA2) to cultured cells was performed as reported [4–6]. Cells were irradiated at desired intensity (30 mJ/cm^2^). Afterwards, cells were returned for incubation in culture medium with indicated treatments.

### Cell viability assay

The cell viability was tested via the MTT assay, which was described in detail in our previous publications [10, 29]. The optical density (OD) value (at 590 nm) of group with treatment was expressed as the percentage of that of the untreated control group [10].

### Trypan blue staining of “dead” RPE cells

Following treatment of cells, trypan blue staining was performed to indentify the “dead” RPE cells (Trypan blue positive). The percentage (%) of trypan blue cells was calculated via an automated cell counter (Merck Millipore, Shanghai, China).

### Cell apoptosis assays

Apoptosis assays, including the Annexin V fluorescence-activated cell sorter (FACS) assay, Histone DNA apoptosis enzyme-linked immunosorbent assay (ELISA) assay and caspase-9 activity assay were described in detail in our previous studies [6, 10, 19, 20, 30]. For FACS assay, Annexin V^+/+^/ PI^−/−^ cells were marked as early apoptosis cells [10, 30]. Annexin V^+/+^/ PI^+/+^ cells were marked as late apoptosis cells [10, 30].

### ROS assay

As previously reported [10, 17], following treatment of cells, the ROS content was measured by the carboxy-H2DCFDA (D399, Invitrogen, Shanghai, China) assay. RPE cells were stained with 1 μM of carboxy-H2-DCFDA at 37°C for 30 min. Cells (1*10^6^) were tested via flow cytometry (BD bioscience). Relative H2DCFDA intensity (vs. untreated control cells) was recorded to reflect cellular ROS content [10, 17].

### Real-time quantitative PCR analysis

Total RNA was extracted using Trizol reagents (15596018, Invitrogen). Five hundred ng of DNA-free total RNA was utilized to perform the reverse transcription with the 2-step RT-PCR kit (Takara Bio Inc., Japan) [10]. The PCR reaction mixture contained 1× SYBR Master Mix (Applied Biosystem, 4309155, Foster City, CA), 500 ng RNA along with 200 nM primers. The ABI Prism 7300 Fast Real-Time quantitative PCR system (Shanghai, China) was utilized for PCR reactions. The ^ΔΔ^Ct method was applied to quantify mRNA expression Glyceraldehyde-3-phosphate dehydrogenase (GAPDH) was tested as an internal control. Primers were described in our previous studies [6, 10] and in published literatures [31].

### Western blot assay

The detailed protocol of Western blot assay was described in our previous studies [5, 10, 20]. For data analysis, each band was quantified and normalized to the indicated loading control via the ImageJ software [5]. For the Western blot assay, each lane was loaded with exact same amount of quantified protein lysates (30 μg per sample). Same set of lysate samples were run in sister gels to test different proteins.

### Detection of mitochondrial depolarization (ΔΨm)

As described in our previous study [32], the cell mitochondrial membrane potential (MMP) reduction, an indicator of mitochondrial depolarization, was measured via JC-1 dye (T3168, Invitrogen, Shanghai, China). With the MMP decreasing, monomeric JC-1 will be formed in the cytosol, exhibiting green fluorescence. Following applied treatment, cells were stained with 5 μg/mL of JC-1 for 10 min at 37°C. Afterwards, cells were washed and tested on a microplate reader with an excitation filter of 485 nm and emission filter of 527 nm. Fluorescence intensity was recorded as the indicator of mitochondrial depolarization.

### shRNA knockdown

Nrf2 shRNA-1 (sc-37030-V), Akt1 shRNA (sc-29195-V) and scramble control shRNA lentiviral particles were purchased from Santa Cruz Biotech (Santa Cruz); Lentiviral Nrf2 shRNA-2, with non-overlapping sequence with Nrf2 shRNA-1, was designed and verified by Genechem (Shanghai, China). The lentiviral particles (10 μL/mL) were added directly to cultured ARPE-19 cells for 36 h. Cells were then subjected to puromycin (1.0 μg/mL) selection for 8–10 passages until resistant colonies can be identified. The expression of targeted protein (Nrf2 or Akt1) in stable cells was always tested by Western blot assay.

### Nrf2 mutation

The S40T dominant negative (“DN”) Nrf2 pSV2 puro Flag plasmid was described previously [6]. The DN-Nrf2 plasmid or the empty vector (pSV2 neo) was transfected to ARPE-19 cells via Lipofectamine 2000 protocol (Invitrogen, Shanghai, China). Stable ARPE-19 cells expressing DN-Nrf2 or the vector were selected via puromycin (2.5 μg/mL) [6].

### Mouse retinal light damage and electroretinography (ERG) recording

The weight-matched male BALB/c mice (6–8 week old) were utilized. The retinal light damage model was described in detail in our previous studies [6]. Briefly, mice were kept in dark for 24 h. Afterwards, one drop of 0.5% tropicamide and 0.5% phenylephrine hydrochloride were added for pupillary dilation. The mice retina were then exposed to white fluorescent light (5000 lux) [6, 23]. To evaluate the *in vivo* activity of SC79, 30 min before light exposure, SC79 (at 50 ng/eye, in PBS) was injected intravitreally to the left eyes. For detecting ERG [6, 23], twenty-four h after light exposure, a single light-flash eye stimulus (3000 cd/m^2^ for 10 ms) was applied [6]. This ERG recording process was conducted under dim red light, and the mice were kept warm during the process. The a-wave and b-wave amplitudes were measured as described [6]. All protocols were approved by the IACUC and Ethical Committee of all authors institutions.

### Statistical analysis

Quantitative results were normalized to the control values of each assay, and were presented as mean ± standard deviation (SD). Data were analyzed by one-way ANOVA followed by a Scheffe's *f*-test via SPSS 18.0 software (SPSS Inc., Chicago, IL). Significance was chosen as *p* < 0.05

## SUPPLEMENTARY MATERIALS FIGURE


