# Supplementary material for: SC79 protects retinal pigment epithelium cells from UV radiation via activating Akt-Nrf2 signaling
Source: Oncotarget. 2016 Aug 9;7(37):60123–32. doi: 10.18632/oncotarget.11164 (PMC5312373; doi:10.18632/oncotarget.11164)
Supplement: Supplementary file 1 [file oncotarget-07-60123-s001.pdf]

SC79 protects retinal pigment epithelium cells from UV radiation via activating Akt-Nrf2 signaling

Supplementary Materials

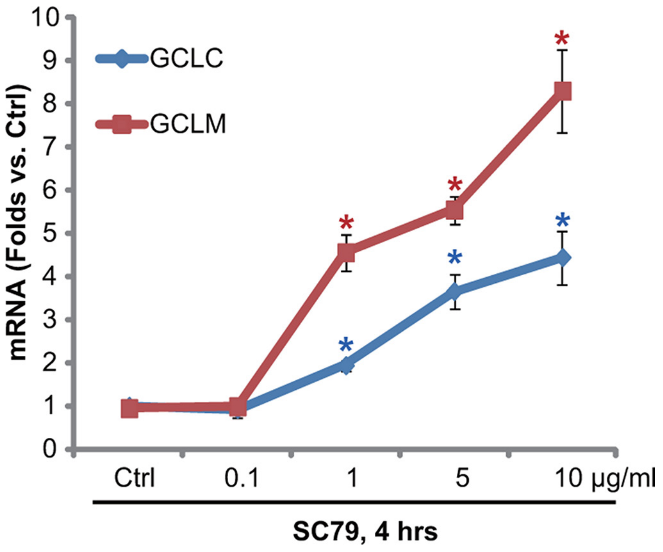

Supplementary Figure S1: Relative mRNA expression of listed genes [γ-glutamyl cystine ligase catalytic subunit (GCLC) and γ-glutamyl cystine ligase modifying subunit (GCLM)] in SC79-treated APRE-19 cells was shown. For each assay, *n* = 5. **p* < 0.05 vs. “Ctrl” group.
